# The Comparative Immunotropic Activity of Carrageenan, Chitosan and Their Complexes

**DOI:** 10.3390/md18090458

**Published:** 2020-09-04

**Authors:** Viktoriya N. Davydova, Irina V. Sorokina, Aleksandra V. Volod’ko, Ekaterina V. Sokolova, Marina S. Borisova, Irina M. Yermak

**Affiliations:** 1 G.B. Elyakov Pacific Institute of Bioorganic Chemistry, Far Eastern Branch, Russian Academy of Sciences, Prospect 100 let Vladivostoku 159, 690022 Vladivostok, Russia; morskaia@list.ru (A.V.V.); eka9739@yandex.ru (E.V.S.); imyer@mail.ru (I.M.Y.); 2 N.N. Vorozhtsov Novosibirsk Institute of Organic Chemistry, Siberian Branch of the Russian Academy of Sciences, Lavrentjev Ave. 9, 630090 Novosibirsk, Russia; sorokina.irina55@gmail.com (I.V.S.); mborisova@nioch.nsc.ru (M.S.B.)

**Keywords:** carrageenan, chitosan, polyelectrolyte complex, cytokine, nitric oxide, anti-inflammatory activity

## Abstract

The immunotropic activity of polyelectrolyte complexes (PEC) of κ-carrageenan (κ-CGN) and chitosan (CH) of various compositions was assessed in comparison with the initial polysaccharides in comparable doses. For this, two soluble forms of PEC, with an excess of CH (CH:CGN mass ratios of 10:1) and with an excess of CGN (CH: CGN mass ratios of 1:10) were prepared. The ability of PEC to scavenge NO depended on the content of the κ-CGN in the PEC. The ability of the PEC to induce the synthesis of pro-inflammatory (tumor necrosis factor-α (TNF-α)) and anti-inflammatory (interleukine-10 (IL-10)) cytokines in peripheral blood mononuclear cell was determined by the activity of the initial κ-CGN, regardless of their composition. The anti-inflammatory activity of PEC and the initial compounds was studied using test of histamine-, concanavalin A-, and sheep erythrocyte immunization-induced inflammation in mice. The highest activity of PEC, as well as the initial polysaccharides κ-CGN and CH, was observed in a histamine-induced exudative inflammation, directly related to the activation of phagocytic cells, i.e., macrophages and neutrophils.

## 1. Introduction

Natural polysaccharides are promising compounds for use in biomedicine and pharmaceuticals. The main prerequisites for this are the combination of their abundance and simplicity of preparation with biocompatibility and a broad spectrum of biological effects, such as immunostimulating, antioxidant, antitumor, antimicrobial, and antiviral.

One of the most well-known polysaccharides of red algae is carrageenan (CGN). CGN is a sulfated galactose copolymer composed of alternating units of D-galactose and 3,6-anhydro-galactose joined by α-1,3 and β-1,4-glycosidic linkages [1]. CGN is classified into various types such as λ, κ, ι, ε, μ, depending on the amount and location of sulfate groups as well as the presence or absence of 3,6-anhydro-galactose units [2]. CGNs have diverse activities including immunomodulatory [3], anticoagulant [4], antithrombotic [5], antiviral [6], and antitumor effects [7]. In recent years, CGNs have been increasingly used for pharmaceutical purposes. CGNs are one source of soluble dietary fibers [8]. Standard animal safety studies in which CGN was administered in diet showed no adverse effects [9]. Due to their biocompatibility, safety (USP35-NF30S1, BP2012, EP7.0), availability, wide range of biological activity, a simple thermo-reversible gelation mechanism, viscoelastic properties and the ability to form complexes with polycations via electrostatic interactions, CGNs are ideal components to obtain new vehicles for the delivery therapeutic substances that can be retained at mucosal surfaces and release the drug slowly [10].

Chitosan (CH) is a natural polycation, β-1,4-linked glucosaminoglycan, which have some acetylated amino groups. CH is a non-toxic, biodegradable and non-immunogenic agent [11] used widely as a biomaterial with an established safety profile in humans, as pharmaceutical excipient, weight loss supplement [11], and as a major component of hemostatic dressings [12]. At relatively low pH (<6.5), chitosan is positively charged and soluble in dilute aqueous solutions [13] CGN.

Marine polysaccharides stimulate different types of immune system cells, both in vitro and in vivo, to produce and secrete molecules with immunostimulatory effects [14,15]. CGNs have been demonstrated to play an important role as free-radical scavengers and antioxidants for the prevention of oxidative damage in living organisms [16]. Our studies on the antioxidant capacity of CGN [17] have shown that the activity of carrageenans depends on the polysaccharide structure. Hence, they have therapeutic potential for the treatment of immunological disorders.

Inflammation is the first biological response of the immune system to infection or irritation. However, in some cases, it can become chronic, and lead to tissue damage [18]. Non-steroidal anti-inflammatories drugs are often used to treat of inflammation. However, their prolonged usage is followed by complications, including damage to the gastrointestinal tract and other side effects [19]. Therefore, the search for new bioactive compounds with anti-inflammatory activities with minimal adverse effects is of great importance. CGN. We have shown that chitosan with molecular weight of 110 kDa ex vivo inhibits the synthesis of anti-inflammatory cytokine, the tumor necrosis factor alpha (TNF-α) induced by endotoxin and stimulates synthesis of the anti-inflammatory cytokine interleukene (IL-10) by oral administration in the blood serum of mice [20]. We have also determined that κ-CGN [21] and chitosan [20] possess anti-inflammatory activity in a model of acetic acid induced colitis in mice. Recently, we have shown the inhibitory effect of CGNs on inflammation caused by endotoxin [22].

The mechanism of action of CGNs is not yet completely understood; however, dietary studies done in animals with food-grade CGN shown no intestinal inflammation [23]. There are some conflicting reports on the effects of CGN on the gastrointestinal tract what, may be in part due to the fact that a food-grade CGN is often confused with a degraded carrageenan, termed poligeenan [24]. In contrast to CGN, poligeenan is not produced biologically but only in the laboratory or commercially by subjecting CGN to very low pH at 0.9–1.3 and non-physiological temperatures of >80 °C for several hours [24]. High molecular weight or food-grade CGN is ingested at low dosages; is not absorbed across the intestinal epithelium, is wholly excreted in feces, does not enter the systemic circulation, and does not cause intestinal ulceration or inflammation. The safety of CGN is supported by a large number of animal oral safety studies in which no adverse effects were reported at high doses (up to 5% in diet) [9].

The obtaining of polyelectrolyte complexes (PEC) by binding two opposite charged polymers is the way to improve and expand the range of properties of polyionic polysaccharides. This allows the modification of their functional properties to improve stability and facilitate application. For example, stable forms of PEC CH-CGN can be obtained in soluble form, in the form of gels, films, sponges, and microparticles. PEC are promising materials for use in biomedicine and pharmaceuticals, since the combination of the favorable properties of each constituent polymer leads to new systems with improved properties, which quite often substantially differ from those of the individual polymers [25]. These properties can be modulated by adjusting the conditions of complex formation, i.e., varying pH, ionic strength, polysaccharide concentration, ratio of the biopolymers, and temperature [26]. In addition, PEC can easily adapt to the requirements dictated by specific applications, for example, by changing the composition of the monomer, the density of the formed bonds, particle size, surface charge density, or type of solvent [27]. We can expect synergies between the components of the complex, the predominance of the activity of one of the components, or the loss of one or another activity of the starting polyions. Although the biological activity of polysaccharides has been actively studied, there is very little data on the physiological effects of complexes based on them.

We previously studied the conditions for the formation of a soluble form of PEC and found that the complexes have gastroprotective [28] and antibacterial [29] activity. The activity of the complexes in these tests exceeded the activity of the starting components [28].

The aim of this work was to study the anti-inflammatory activity of PEC, consisting of various compositions of κ-CGN and CH, in comparison with the initial polysaccharides taken in comparable doses. Tests of acute inflammation in mouse paw associated with the induction of a predominantly cellular immune response mediated by phagocytes (histamine test), B-lymphocytes (concanavalin A test) or T-lymphocytes (HRT reaction) were used. In ex vivo experiments, the effect of agents on the cytokine profile of donated blood was also evaluated.

## 2. Results

### 2.1. Characterisation of the Initial Polysaccharides

For the study of the anti-inflammatory activity of the polysaccharides, κ-CGN, CH and soluble forms of their PEC were prepared. κ-CGN from red algae *Chondrus armatus* was obtained as described earlier, it structure was determined according to [30]. The molecular weight (MW) of κ-CGN was determined by viscometry and was found to be 250 kDa.

CH was obtained by the alkaline deacetylation of crab chitin as described in [31]. The MW determined by viscometry was 110 kDa, and the deacetylation degree (DA), determined according [32] by FTIR-spectroscopy was 96%.

### 2.2. Characterization of PEC κ-CGN-CH

Activity of biopolymers is determined by numerous parameters, among which are the size of the molecule, its charge, density, solubility, the molecule conformation and flexibility of polysaccharide chain. The formation of the complex affects all these parameters; it is obvious that the activity of the complexes will differ from that of the original compounds.

The process of obtaining complexes of CH with CGN in soluble form was studied by us in detail earlier. It was shown that soluble complexes were obtained preferentially by mixing the starting components at given ratios with an excess of one of the components [33]. The complex formation of κ-CGN:CH 1:10 and 10:1 *w*/*w* was shown by centrifugation in a Percoll gradient [34].

**Figure 1:**
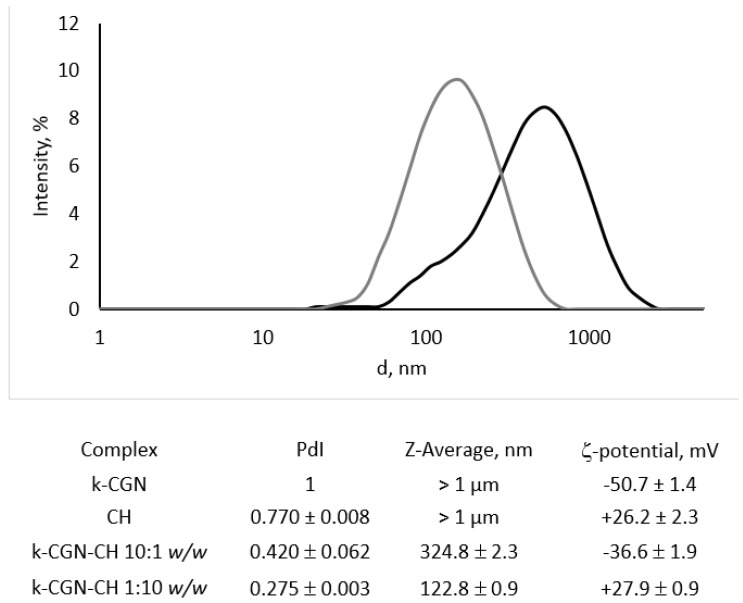
Intensity particle size distribution of κ-CGN-CH 10:1 *w*/*w* (black line) and κ-CGN-CH 1:10 *w*/*w* (grey line) complexes measured on a Nano ZS (Malvern PANalytical, Malvern, UK) using scattering detection at 173°. The hydrodynamic diameters of the particles were automatically calculated with the instrument’s software based on analysis of the autocorrelation function.

In this study the soluble PEC with a κ-CGN:CH weight ratios of 10:1 and 1:10 were prepared by mixing solutions of the initial polysaccharides and characterized by dynamic light scattering (Figure 1).

The initial CH and κ-CGN presented as polydisperse particles, heterogeneous in size, with Z-average of about 1 µm (data not shown), which corresponds to their polysaccharide nature [35]. The nature of polysaccharides creates certain difficulties for their study by hydrodynamic methods. The mutual repulsion of charged groups along the polymer chain is the reason why the molecule becomes strongly extended, while its hydrophilic part is in the hydrate shell. This makes it difficult to study by the DLS (dynamic light scattering) and we could not obtain a size distribution for CGN and CH with an acceptable value of polydispersity index PdI. The surface potential of CH was +26.2 ± 2.3 mV, while that of κ-CGN was −50.7 ± 1.4 mV.

After complex formation, the polydispersity of the system was reduced significantly. The complex with an excess of CH (κ-CGN-CH 1:10 *w*/*w*) was fairly composed of homogeneous, positively charged particles with an average diameter of about 123 nm. Negatively charged particles of the complex with an excess of κ-CGN (κ-CGN-CH 10:1 *w*/*w*) was more polydisperse in size with an average diameter of about 325 nm.

The charge of the PEC was determined by the polymer, that was in excess. The charge of CGN in the complex with its excess (κ-CGN-CH 10: 1 *w*/*w*) was partially neutralized, but the charge of the complex with an excess of CH (κ-CGN-CH 1:10 *w*/*w*) corresponded to the charge of the initial polycation, which may indicate surface localization of CH in the complex, shown by us earlier [29].

### 2.3. In Vitro Activity of the Initial Polysaccharides and Their Complexes

In biomedicine, much attention has been paid to natural antioxidants and their association with health benefits [36]. Taking into account the complexity of antioxidants action in vivo, different in vitro methodologies have been developed to estimate, in a simple experimental way, the capacity of antioxidants and their complex mixtures to neutralize the reactive oxygen and nitrogen species (ROS/RNS) [37].

**Figure 2:**
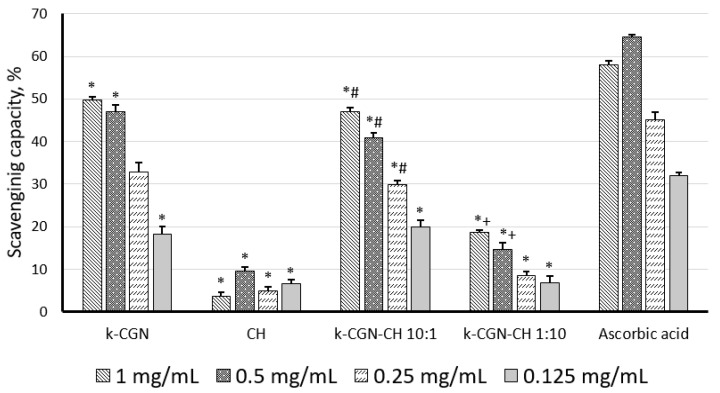
Nitric oxide scavenging effect of κ-carrageenan (κ-CGN), chitosan (CH) and their complexes. Statistical analysis was done by one-way ANOVA (analysis of variance). *—Differences between samples and the control were significant, *p* < 0.05; #—Differences between polyelectrolyte complexes (PEC) and κ-CGR were significant, *p* < 0.05; +—Differences between PEC and CH were significant, *p* < 0.05.

We considered the potential antioxidant activity of κ-CGN, CH, and PEC determined by their ability to bind nitric oxide (NO) (Figure 2).

According to Figure 2, κ-CGN showed a pronounced ability to scavenge NO, and its activity (at 1 mg/mL, 0.5 mg/mL) was comparable to the action of the standard (ascorbic acid). CH did not show the ability to scavenge NO (Figure 2). The low activity of this polysaccharide in this test was noted earlier [38]. It was previously reported that with an increase in the MW of chitosan up to 100 kDa, its antioxidant activity have decreased [39]. Together with this, the CH used here had a MW of 110 kDa. The PEC with a high content of κ-CGN exhibited activity comparable to the activity of the initial κ-CGN. Unlike the initial polycation, the complex with a high CH content (κ-CGN-CH 1:10 *w*/*w*) exhibited antioxidant activity, although it was less pronounced than κ-CGN and the complex κ-CGN-CH 10:1 *w*/*w*.

### 2.4. Ex Vivo Activity of the Initial Polysaccharides and Their Complexes

**Figure 3:**
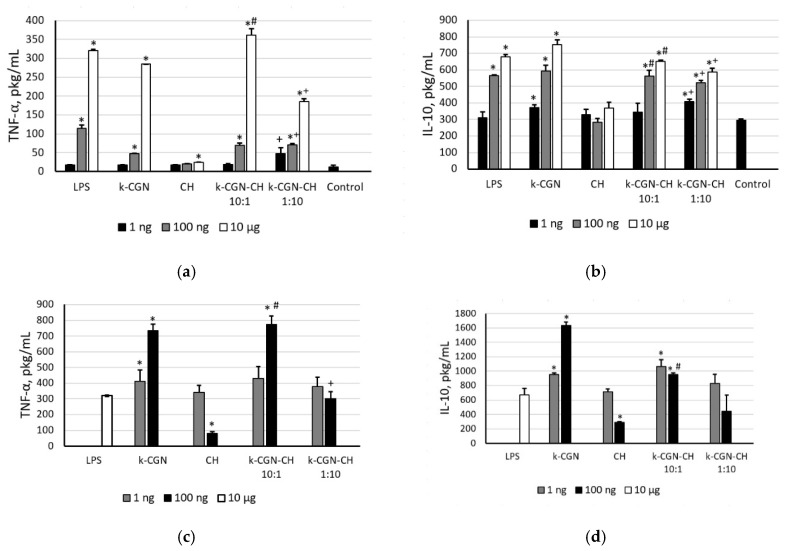
(**a**) Tumor necrosis factor-α (TNF-α) and (**b**) interleukine-10 (IL-10) level stimulated by κ-CGR, CH and complexes. TNF-α (**c**) and IL-10 (**d**) level stimulated by preliminary incubation of peripheral blood mononuclear cell (PBMCs) with *Escherichia coli* LPS (10 min), then by κ-CGN, CH, and complexes. Contents of a cytokine in serum are presented as a mean ± SD. Whole blood samples were obtained from 5 healthy subjects and incubated with the polysaccharides and PEC at different concentrations. The cytokine level in serum of normal donors (**a**,**b**) or the cytokine level in serum after preliminary incubation of PMBC with *E. coli* LPS (**c**,**d**) were considered as a negative control used for statistical calculation. Statistical analysis was done using one-way ANOVA. *—Differences between samples and the control were significant, *p* < 0.05; #—Differences between PEC and κ-CGR were significant, *p* < 0.05; +—Differences between PEC and CH were significant, *p* < 0.05.

The ability of polysaccharides and their complexes to induce the synthesis of pro-inflammatory cytokines, i.e., tumor necrosis factor-α (TNF-α), and anti-inflammatory cytokine, i.e., interleukine-10 (IL-10), in peripheral blood mononuclear cell (PBMCs) was studied. Both of these cytokines play important roles in the immune response, as they activate circulating cells and stimulate the production of chemokines and adhesion molecules [40]. Figure 3 shows that the ability of the polysaccharides and their PEC to activate cells and to induce cytokine synthesis has been correlated to the activity of LPS. At a high concentration (10 µg/mL) κ-CGN caused a strong increase in the level of cytokines, in comparison with the control, while at a low concentration (1–10 ng/mL) the activity was insignificant. CH was inert in this test.

The CGN and PEC did not show an anti-inflammatory activity in the presence of LPS (Figure 3c,d). Only chitosan at a concentration of 100 ng/mL had the ability to suppress of LPS-induced TNF-α and IL-10 production.

According to Figure 3, the ability of PEC to induce the synthesis of pro-inflammatory (TNF-α) and anti-inflammatory (IL-10) cytokines in the whole blood cell assay in both conditions (with and without LPS) is determined as the activity of the initial κ-CGN, regardless of their composition. The effect of the complex with an excess of CGN was similar to that the initial κ-GRG. The activity of the complex with an excess of CH was slightly lower than that of the initial κ-GRG, that possibly due to the presence of CH.

### 2.5. In Vivo Anti-Inflammatory Activity of the Initial Polysaccharides and Their Complexes

The anti-inflammatory activity of PEC and the initial compounds was evaluated using histamine-, concanavalin A- and sheep erythrocyte immunization-induced paw edema in mice. The results are expressed as the inflammatory edema index (IEI) and percentage of anti-inflammatory activity (AIA) relative to the control group (Table 1).

In experiments in vivo, polysaccharides and complexes were administered intraperitoneally in the form of aqueous solutions to provide more efficient delivery of compounds. The doses of the complexes were selected taking into account their low toxicity and the ability to dissolve in the volume of the solvent (water) allowed for intraperitoneal administration to mice (0.2 mL/10 g of body weight). Doses of polysaccharides were taken in accordance with their maximum proportion in the composition of the complexes.

The data indicate that preventive i.p. injections of PEC and both polysaccharides significantly decreased local inflammation induced by histamine in mice. CH and PEC κ-CGN:CH 1:10 demonstrated the most potent decrease IEI values relative to the control group (1.8 and 1.7 times, correspondingly), which are insignificantly different from that of indomethacin (2.3 times) (Table 1). κ-CGN alone and PEC κ-CGN:CH 10:1 were less active in decreasing edema (1.4 and 1.3 times, correspondingly)

The results presented in Table 1 show a downward trend in activity of tested agents with higher level of the κ-CGN. It correlates with the data from the ex vivo experiments, indicating an increase in TNF-α production at a high dose of κ-CGN. TNF-α is a pro-inflammatory cytokine secreted by activated macrophages in an early stage of acute inflammation. The low level of TNF-α indicates attenuation of the inflammatory reaction in response to a low dose of κ-CGN.

In the concanavalin A inflammation test, κ-CGN and CH showed a moderate anti-inflammatory effect (lowering the edema index by 1.9 and 1.7 times, respectively, versus control) in comparison with diclofenac (2.6 times), while the complexes were not active (Table 1). In the DTH reaction, only κ-CGN alone inhibits inflammation. Both PECs and CH were inactive (Table 1).

## 3. Discussion

Polysaccharide PEC are well-tolerated macroorganism systems and can be used in various fields, such as drug delivery systems, cell cultivation and enzyme immobilization, or tissue reparation and regeneration [41]. However, despite the numerous studies devoted to the preparation and application of PEC, their biological activity and the modification of the activity of the initial polysaccharides after PEC formation have been studied insufficiently.

CH and CGN have pronounced immunostimulatory properties [1,20,34,35,36]. Therefore, this study was aimed at the comparative analysis of the effects of PEC and initial compounds on some components of the immune system associated with immunomodulating activity.

Immunity is the host physiological defense of an organism against pathogenic factors such as toxins, infectious microorganisms and viruses. The immune response passes on the collective and coordinated cooperation of specific cells and humoral mediators including vascular and cell adhesion molecules and proteins of the complement system. The most relevant cells involved in the immune response are lymphocytes, monocytes/macrophages, polymorphonuclear granulocytes, and mast cells. Macrophages and antigen-presenting cells (APC) are involved in all stages of non-specific immune responses and are responsible for phagocytosis, antigen processing and presentation, secretion of NO and cytokines such as and TNF-α, as well as antibody-dependent and cell-mediated cytotoxicity [42]. The effect of complexation on some cellular immunity reactions mediated by B- and T-lymphocytes in vitro, ex vivo and in vivo models have been considered.

In this study a comparative analysis of the immunotropic activity of polysaccharides and their complexes was carried out. The soluble complexes obtained by mixing the starting components at given ratios of κ-CGN-CH 1:10 *w*/*w* or κ-CGN-CH 10:1 *w*/*w*. The complexes formation was accompanied changes in the physicochemical parameters of the initial polysaccharides. After complex formation, the polydispersity of the initial polysaccharides was reduced significantly (Figure 1). According to our earlier data [15] the macromolecular structure of PEC was different from the structures of the initial components in the appropriate concentrations. The mechanism of PEC complex particle formation is determined by the nature of the prevailing polymer. The particles in the soluble complexes κ-CGN-CH 1:10 *w*/*w* has surface localization of the polycation and stabilized by unreacted CH amine groups. The incorporation of CH into the network structure of CG for complexes κ-CGN-CH 10:1 *w*/*w* was assumed [15].

NO is a highly reactive free radical involved in a number of physiological and pathological processes [43]. NO acts immunologically as a cytotoxic agent on invading microorganisms in macrophages or on tumor cells [44]. Our studies on the antioxidant capacity of CGN [17] and studies on other polyionic polysaccharides have shown that antioxidant activity is determined by the location of ionic groups in the carbohydrate chain and by the molecular weight of this chain [38,45]. According to our results, the activity of PEC to scavenge of NO depends on the content of κ-CGN in the complex: PECs with a high content of κ-CGN exhibited activity comparable to the activity of the κ-CGN alone.

Sulfated polysaccharides are commonly reported as strong antioxidants, which is partly due to its ordered, extended structure. Sulfated polysaccharides usually trap free radicals in an electrostatic manner since the sulfate groups usually generate a highly acidic environment and the sulfur substitution may also weaken hydrogen bond interactions between polysaccharides [46]. This explains the high activity of the initial κ-CGN and the PEC κ-CGN:CH 10:1 *w*/*w*. It is noteworthy that the complex with a high content of CH, which, according to our early data [29], had surface localization of the polycation, also showed a low ability to scavenge NO, unlike the initial CH.

As already described in the literature, polysaccharides have an important immunostimulatory capacity through the production of cytokines [8,9,16]. The data presented in this study clearly demonstrate that κ-CGN and PEC at a low concentration (1 ng/mL) do not induce the pro-inflammatory TNF-α, which is an important mediator for the induction of many biological responses [47]. The initial CH did not have the ability to induce cytokine synthesis, but the PEC κ-CGN:CH 1:10 *w*/*w* was able to induce the synthesis of TNF-α and IL-10 in cells, although to a lesser extent than the original κ-CGN. Du et al. [48] suggested that the solubility and assembly of the components influence the immuno-pharmacological activities of glucans. Wang et al. [49] postulated that the relatively high bioactivity of polysaccharides can be attributed to good water solubility and expanded chain conformation. So, it can be assumed, that κ-CGN-CH 1:10 *w*/*w* PEC, with a small particle size and low polydispersity detected by dynamic light scattering (Figure 1) can facilitate contact with the cell compared to the initial more heterogeneous and tightly packed CH.

The results of this investigation indicate that PEC has anti-inflammatory activity against phagocytes, activated by histamine (Table 1). Histamine is the main mediator of the initial phase of acute inflammation. Intra-plantar injection of histamine induces the activation of resident macrophages and mast cells in the tissue of the mouse paw. The pro-inflammatory effects of histamine are also achieved through interactions with its receptors on the endothelium and blood leukocytes. Binding H1 receptors causes an increase in vascular permeability, as well as the formation of prostaglandin E2 (PGE2), leukotrienes and chemokines, which attract eosinophils and neutrophils to the site of inflammation [50]. Preventive i.p. administration of CH, κ-CGN and their water-soluble PEC of the 1:10 and 10:1 *w*/*w* compositions significantly delayed the development of acute local inflammation. Under these conditions, their activity was comparable to non-steroidal anti-inflammatory drugs, the effect of which is based on the inhibition of cyclooxygenase 1 and 2 on the membranes of endothelial and blood cells, followed by impaired synthesis of prostaglandins E2 and F2α, thromboxane A2, prostacyclin, leukotrienes, and the release of lysosomal enzymes. It can be assumed that polysaccharides use a similar mechanism as a result of direct contact with the membranes of effector cells. This is confirmed by our recent data on the ability of CGN to inhibit of PGE2 synthesis at low concentrations [51]. On the other hand, the scavenger abilities of κ-CGN and its water-soluble PEC could attenuate damage associated with highly active NO-species, which are produced by phagocytes, as shown in ex vivo experiments described here (Figure 2).

The inflammatory response induced by concanavalin A is an example of type I or immediate hypersensitivity, which is due to IgE antibody production and the development allergic sensitization. This type of inflammation is associated with the activation of Th2 cells and adaptive immunity. The polyvalent lectin concanavalin A stimulates the release of histamine and leukotrienes from mast cells and basophils, activates neutrophils, increases the activity of cyclooxygenase-2 (COG-2) and stimulates the proliferation and differentiation of Th2 lymphocytes. Lectin-activated cells secrete various inflammatory mediators, quickly causing severe edema and inflammation in the form of an allergic-like reaction, but without contact with a native allergen [52,53,54]. Intraperitoneal administration of CH and κ-CGN reduced mast cell degranulation and the subsequent development of inflammation. However, the PEC of the 1:10 and 10:1 *w*/*w* compositions had no anti-inflammatory activity. It is suggested that free CH and κ-CGN could occupy the membrane surface of mast cells and interrupt the binding of lectin molecules, or could block the transfer of pro-inflammatory signals from primary activated mast cells to the membrane receptors of effector cells (macrophages, neutrophils, or lymphocytes), thereby inhibiting downstream cytokine secretion and inflammation.

A similar effect was found with the DTH test which is based on T-cell immunity. In this case, T-lymphocytes, preliminary primed with antigen, stimulate the blast transformation and proliferation of cytotoxic T-lymphocytes. The secreted pro-inflammatory cytokines secreted by them stimulate the activity of macrophages and cytotoxic lymphocytes, which induce an inflammatory response and the development of a granuloma in the mouse paw [42]. Among the tested samples only κ-CGN alone decreased the DTH reaction, while the others did not (Table 1).

The particle surface charge could potentially control biopolymers binding to tissue both in vivo and in vitro. Cellular surfaces are dominated by negatively charged sulfated proteoglycans, molecules that play pivotal roles in cellular proliferation, migration, and motility [55], therefore, interactions between proteoglycans and positively charged particles are preferred [56]. At the same time negatively charged nanoparticles (NP) show highly increased bioadhesive properties and are absorbed by both M cells and absorptive enterocytes [57]. Jung et al. 57] has also shown that charges on the NP surface are not the only requirement, a combination of both NP surface charges and increased hydrophilicity of the matrix material seem to affect the gastrointestinal uptake in a positive sense. Unfortunately, to date, information on the correlation of activity depending on the charge of natural polysaccharides is very limited. We earlier have shown [29], that CH, CGN and their positively and negatively charged complexes had the different ability to suppress the biofilm formation by Gram-negative and Gram-positive microorganisms. CH and positively charged complexes inhibited only biofilm formation by Gram positive microorganisms (*B. subtilis*). CGN and negatively charged PEC prevented the formation of biofilm by Gram negative microorganisms (*E. coli*) and Gram positive microorganisms.

It could be expected that the activity of the studied PEC would be determined by the prevailing polysaccharide. However, only in the in vitro test, we observed some dependence of the ability to scavenge NO on the polysaccharide content in the PEC. In ex vivo test which is more complex systems, including not only cells but also different soluble serum components, the direct dependency of the activity of the PEC on the polysaccharide contents was not observed. It is much more difficult to carry out any correlations between the activity and surface characteristics of the objects in in vivo experiments. The overall protein concentrations in typical body fluids (e.g., blood, lung, gut) and intracellular environments can be as high as 0.35 gmL^−1^ [58]. These fluids may contain more than 3000 different proteins at widely differing concentrations. It was shown that nanomaterials adsorb biomolecules on contact with biological fluids [59]. Thus the components of the biological fluids can affect the manifestation of a biological activity of studied compounds and an activity determined in experiments in vitro and in vivo can manifest itself in different ways.

A comparison of in vivo test results suggests that the immunomodulatory effect of polysaccharides and the PEC was directly related to their ability to bind to the membrane receptors of immune cells and to influence signal transmission between components of the inflammatory process. The greatest activity of PEC, as well as the initial polysaccharides κ-CGN and CH, was observed in acute exudative inflammation, directly related to the activation of phagocytic cells, i.e., macrophages and neutrophils. The macrophage surface bears pattern recognition receptors (PRRs), which can recognize and bind the molecules of bacterial polysaccharides [42]. Moreover several members of the TLR (toll-like receptor) family interact with exogenous peptidoglycans and polysaccharides to induce adaptive immunity and modulate immune response [60]. However, in tests mediated by B- and T-lymphocyte stimulation, PEC did not show activity, unlike the free polysaccharides. Thus, the complexation of the CH and κ-CGN led to a decrease in their immunological activity in terms of B- and T-cell immunity. It is assumed that this is due to specific physicochemical limitations that impede PEC interactions with membrane receptors on lymphocytes.

## 4. Materials and Methods

### 4.1. Polysacharides

A CH sample with molecular weight of 110 kDa and 6% degree of N-acetylation was obtained by alkaline treatment of crab chitin according to the published protocol [31]. It was deacetylated with the mixture of 40% aqueous solution of NaOH with isopropyl alcohol (1:16 *v*:*v*) under heating for 7 h at 100 °C. The pellet was filtered and dissolved in water acidified with hydrochloric acid (pH 3.5), dialyzed against water, and lyophilized [61].

The sample of κ-CGN was isolated by extraction with hot water from the red algae *Chondrus armatus* (Gigartinaceae). The algae were collected at Peter the Great Bay (Sea of Japan) washed with tap water in order to remove excess of salt. Dried and milled algae (50 g) were suspended in hot water (1.5 L) and the polysaccharides were extracted at 90 °C for 2 h in a water bath. The polysaccharides were fractionated into gelling KCl-insoluble and non-gelling KCl-soluble fractions and their structures were established according to a published protocol [62]. KCl-insoluble fraction, which according to the structural analysis was assigned to k-CGN have been used.

### 4.2. Complexes CGN:CH

The complexes of κ-CGN with CH were prepared by mixing solutions of the initial components at the given ratios as described in [33]. The concentration of the component in excess in the complex for all experiments was 0.1 mg/mL. The mixture was incubated for 15 min.

### 4.3. Dynamic Light Scattering (DLS) and Electrophoretic Properties of the CGN:CH Complexes

The sizes and ζ-potentials of the initial polysaccharides and their PECs in solution were determined using a ZetaSizer NanoZS system (Malvern PANalytical, Malvern, UK) operating at 633 nm. The measurements were performed at 25 °C. The hydrodynamic diameters of the particles were automatically calculated with the instrument’s software based on analysis of the autocorrelation function. The ζ-potentials were calculated from the experimentally determined.

### 4.4. Determination of NO Scavenging Capacity (Microplate)

The reaction mixture containing sodium nitroprusside (10 mM, 75 µL) in phosphate buffered saline (PBS) and samples or reference compound were incubated at 25 °C for 150 min. Then, the Griess reagent (1% sulfanilamide, 0.1% naphthylethylene diamine dihydrochloride in 2% H_3_PO_4_) was added to the reaction mixture (1:1 = *v*:*v*). The absorbance of the chromophore formed during the diazotization of nitrite with sulfanilamide and subsequent coupling with napthylethylenediamine was measured at 546 nm. The percentage inhibition of NO generated was measured by comparing the absorbance values of the control and a sample in quadruplicate. Ascorbic acid was used as a positive control [63].

All data were expressed as mean ± standard deviation. Statistical analysis was done by one-way ANOVA. Differences were considered to be statistically significant if *p* < 0.05.

### 4.5. IL-10 and TNF-α Inducing Activity on the Human Blood Cells

Blood processing was performed using procedure of De Groote et al. [64]. Peripheral blood was collected by vena puncture into sterile siliconized tubes containing 30 IU of lithium heparinate per 5 mL tube diluted 1:5 in sterile Medium 199 (Sigma-Aldrich, Saint Louis, MO, USA) containing 300 mg/L of glutamine (Gibco, Life Technology, Darmstadt, Germany) and 50 µg/mL of gentamicin. Diluted blood (0.1 mL) was transferred into sterile polypropylene plates and then incubated with the samples or with LPS *E. coli* 10 µg (10 min), then with the samples (37 °C, 5% CO_2_). After 24 h the supernatants were collected and frozen followed by cytokine determination using a specific ELISA kit (“Cytokine”, Saint-Petersburg, Russia). The study protocol was approved by the medical ethics committee of the local hospital (Vladivostok, Russian Federation). Informed consent was obtained from all subjects who participated in the study. All donors were free of medicines administration for 14 days prior to blood sampling. Blood was drawn from the antecubital vein of normal healthy human volunteers and anticoagulated in plastic tubes (Greiner Bio-One International AG, Kremsmuenster, Austria) with 30 IU lithium heparinate used as an anticoagulant.

All data were expressed as mean ± standard deviation. Statistical analysis was done by one-way ANOVA. Differences were considered to be statistically significant if *p* < 0.05.

### 4.6. Biological Activity In Vivo

#### 4.6.1. Animals

The biological experiments were carried out on outbred and C57BL/6 male mice housed in standard environmental conditions (room temperature, 12 h light/dark cycle). The animals had free access to standard pellet diet and water ad libitum. All experimental procedures were approved by the Local Bio-Ethical Committee of Medicine Chemistry Department of Novosibirsk Institute of Organic Chemistry SB RAS in accordance with European Communities Council Directive 86/609/EEC.

#### 4.6.2. Anti-Inflammatory Activity

The anti-inflammatory activity of the polysaccharides and their PECs was evaluated on three mouse hind paw edema tests induced by histamine (exudative inflammation), concanavalin A (B-cells dependent inflammation) or delayed type hypersensitivity reaction (T-cells dependent inflammation) [65]. Control and experimental groups consisted of 8-10 animals each. In the histamine and concanavalin A tests of inflammation the substances were injected intraperitoneally (i.p.) in water solutions at doses 10.0 mg/kg (PECs) and 9.0 mg/kg (original κ-CGN or CH). In the test of delayed type hypersensitivity reaction (DTH) the substances were administered intraperitoneally at doses 5.0 mg/kg (PECs) and 4.5 mg/kg (κ-CGN or CH). The last dose values were equal to the highest quota of each polysaccharide in appropriate PEC. The dose of reference substances (indomethacin or diclofenac, “Fluka BioChemica” (Sigma-Aldrich, Saint Louis, MO, USA) was 50 mg/kg [66]. The control group of animals received water. At the end of experiment, the animals were sacrificed by cervical dislocation, the mouse hind paws were cut off at the ankle joint and weighed. The ratio of the difference in weight between the treated and untreated hind paws to the weight of the untreated hind paw was used as an inflammatory edema index (IEI). The anti-inflammatory activity (AIA) was presented as a difference between 100% and percentage of inflammation index versus to the control group.

#### 4.6.3. Histamine-Induced Mouse Paw Edema Test

The outbred male albino mice were injected with 0.05 mL 0.01% histamine in saline solution into the plantar of the hind paw. The collateral paw was injected with saline solution. The tested compounds (κ-CGN, CH, and their PECs) were administered i.p. one hour before the histamine injection. The reference agent indomethacin was administered by the same way at the dose 50 mg/kg. The animals were sacrificed 5 h after the histamine injection.

#### 4.6.4. Concavaline A-Induced Mouse Paw Edema Test

The male mice C57Bl/6 were injected with 0.02 mL 0.5% concanavaline A (Sigma-Aldrich, Saint Louis, MO, USA) in saline solution into the plantar of the hind paw. The collateral paw was injected with saline solution. The tested compounds were administered i.p. one hour before the concanavaline A injection. The reference agent diclofenac was administered by the same way at the 50 mg/kg dose. The animals were sacrificed one hour after the concanavaline A injection.

#### 4.6.5. Delayed Type Hypersensitivity Reaction (DTH) Test

The tested compounds were administered intraperitoneally to the male mice C57Bl/6. One day later the sheep erythrocytes suspension (10^8^ cells) was injected i.p. to control and experimental groups. At the fifth day after immunization the animals were subplantar injected with the sheep erythrocytes suspension (10^8^ cells) into the hind paw. The collateral paw was injected with saline solution. The animals were sacrificed by cervical dislocation 24 h after the last antigen injection

#### 4.6.6. Statistical Analysis

Statistical analyses were performed using “Statistica 6” software. The results of in vivo experiments are given as mean ± SD. The statistically significant difference between groups was calculated by one-way ANOVA by Fisher test (at parametric distribution) or not-parametric Kruskal–Wallis test. A *p*-value of ≤0.05 was considered as statistically significant.

## Figures and Tables

**Table 1:** Anti-inflammatory activity of κ-carrageenan (κ-CGN), chitosan (CH), and their polyelectrolyte complexes (PECs) in mouse paw edema tests.

Agent	Histamine-Induced Inflammation Test		Concanavalin A-Induced Inflammation Test		Delayed Type Hypersensitivity Reaction Test	
	IEI, %	AIA, %	IEI, %	AIA, %	IEI, %	AIA, %
Control	29.6 ± 2.0	0	18.6 ± 2.0	0	14.6 ± 0.6	0
κ-CGN:CH 10:1	22.44 ± 1.53 ** ##	37	20.0 ± 2.3 ##	0	14.6 ± 1.3	0
κ-CGN:CH 1:10	17.45 ± 3.04 **	51	20.9 ± 2.3 ##	0	14.0 ± 1.6	4
κ-CGN	20.77 ± 2.56 **#	37	9.8 ± 1.1 ** #	47	6.4 ± 1.0 ***	56
CH	16.70 ± 3.66 **	53	10.9 ± 1.6 * #	41	15.7 ± 1.7	0
Indomethacin	12.61 ± 1.11 ***	65	−	−	−	−
Diclofenac	−	−	7.2 ± 1.4 ***	61	−	−

* *p* < 0.05, ** *p* < 0.01, *** *p* < 0.001 differences with control group are significant; # *p* < 0.05, ## *p* < 0.001 differences with reference group are significant; IEI—inflammatory edema index, AIA—anti-inflammatory activity. Data represent as mean ± standard error (SEM); *n* = 8 mice in group. Statistical analyses were performed using “Statistica 6” software. The significant difference between groups was calculated by one-way ANOVA by Fisher test (at parametric distribution) or not—parametric Kruskal–Wallis test. A *p*-value of ≤0.05 was considered as statistically significant.

## References

[1] Yermak I.M., Khotimchenko Y.S. (2003). Chemical properties, biological activities and applications of carrageenans from red algae. Recent Adv. Mar. Biotechnol..

[2] Van de Velde F., Knutsen S. (2002). 1H and 13C high resolution NMR spectroscopy of carrageenans: Application in research and industry. Trends Food Sci. Technol..

[3] Yermak I.M., Barabanova A.O., Aminin D.L., Davydova V.N., Sokolova E.V., Solov’Eva T.F., Kim Y.H., Shin K.S. (2012). Effects of structural peculiarities of carrageenans on their immunomodulatory and anticoagulant activities. Carbohydr. Polym..

[4] Sokolova E.V., Byankina A.O., Kalitnik A.A., Kim Y.H., Bogdanovich L.N., Solov’Eva T.F., Yermak I.M. (2014). Influence of red algal sulfated polysaccharides on blood coagulation and platelets activation in vitro. J. Biomed. Mater. Res. Part A.

[5] Ghosh T., Chattopadhyay K., Marschall M., Karmakar P., Mandal P., Ray B. (2009). Focus on antivirally active sulfated polysaccharides: From structure-activity analysis to clinical evaluation. Glycobiology.

[6] Gomaa H.H.A., Elshoubaky G.A. (2016). Antiviral activity of sulfated polysaccharides carrageenan from some marine seaweeds. Int. J. Curr. Pharm. Rev. Res..

[7] Yuan H., Song J., Li X., Li N., Liu S. (2011). Enhanced immunostimulatory and antitumor activity of different derivatives of κ-carrageenan oligosaccharides from Kappaphycus striatum. J. Appl. Phycol..

[8] Lahaye M., Kaeffer B. (1997). Seaweed dietary fibres: Structure, physico-chemical and biological properties relevant to intestinal physiology. Sci. Aliment..

[9] Weiner M.L. (2014). Food additive carrageenan: Part II: A critical review of carrageenan in vivo safety studies. Crit. Rev. Toxicol..

[10] Prajapati V.D., Maheriya P.M., Jani G.K., Solanki H.K. (2014). Carrageenan: A natural seaweed polysaccharide and its applications. Carbohydr. Polym..

[11] Singla A.K., Chawla M. (2001). Chitosan: Some pharmaceutical and biological aspects-an update. J. Pharm. Pharmacol..

[12] Kunio N.R., Riha G.M., Watson K.M., Differding J.A., Schreiber M.A., Watters J.M. (2013). Chitosan based advanced hemostatic dressing is associated with decreased blood loss in a swine uncontrolled hemorrhage model. Am. J. Surg..

[13] Skjak-Braek G., Anthonsen T., Sandford P.A. (1989). Chitin and Chitosan: Sources, Chemistry, Biochemistry, Physical Properties and Applications.

[14] Pérez-Recalde M., Matulewicz M.C., Pujol C.A., Carlucci M.J. (2014). In vitro and in vivo immunomodulatory activity of sulfated polysaccharides from red seaweed Nemalion helminthoides. Int. J. Biol. Macromol..

[15] Liu Q., Xu S., Li L., Pan T., Shi C.L., Liu H., Cao M., Su W., Liu G. (2017). In vitro and in vivo immunomodulatory activity of sulfated polysaccharide from Porphyra haitanensis. Carbohydr. Polym..

[16] Suganya A.M., Sanjivkumar M., Chandran M.N., Palavesam A., Immanuel G. (2016). Pharmacological importance of sulphated polysaccharide carrageenan from red seaweed Kappaphycus alvarezii in comparison with commercial carrageenan. Biomed. Pharmacother..

[17] Sokolova E.V., Barabanova A.O., Homenko V.A., Solov’eva T.F., Bogdanovich R.N., Yermak I.M. (2011). In Vitro and Ex Vivo Studies of Antioxidant Activity of Carrageenans, Sulfated Polysaccharides from Red Algae. Bull. Exp. Biol. Med..

[18] Debnath T., Kim D., Lim B. (2013). Natural Products as a Source of Anti-Inflammatory Agents Associated with Inflammatory Bowel Disease. Molecules.

[19] Bruno A., Tacconelli S., Patrignani P. (2014). Variability in the Response to Non-Steroidal Anti-Inflammatory Drugs: Mechanisms and Perspectives. Basic Clin. Pharmacol. Toxicol..

[20] Davydova V.N., Kalitnik A.A., Markov P.A., Volod’ko A.V., Popov S.V., Ermak I.M. (2016). Cytokine-inducing and anti-inflammatory activity of chitosan and its low-molecular derivative. Appl. Biochem. Microbiol..

[21] Kalitnik A.A., Anastyuk S.D., Barabanova A.O.B., Glazunov V.P., Yermak I.M., Marcov P.A., Popov S.V., Ovodov Y.S. (2015). Gelling polysaccharide from Chondrus armatus and its oligosaccharides: The structural peculiarities and anti-inflammatory activity. Carbohydr. Polym..

[22] Yermak I.M., Volod’ko A.V., Khasina E.I., Davydova V.N., Chusovitin E.A., Goroshko D.L., Kravchenko A.O., Solov’eva T.F., Maleev V.V. (2020). Inhibitory effects of carrageenans on endotoxin-induced inflammation. Mar. Drugs.

[23] McKim J.M., Wilga P.C., Pregenzer J.F., Blakemore W.R. (2015). The common food additive carrageenan is not a ligand for Toll-Like- Receptor 4 (TLR4) in an HEK293-TLR4 reporter cell-line model. Food Chem. Toxicol..

[24] Weiner M.L. (1991). Toxicological properties of carrageenan. Agents Actions.

[25] Matricardi P., Di Meo C., Coviello T., Hennink W.E., Alhaique F. (2013). Interpenetrating polymer networks polysaccharide hydrogels for drug delivery and tissue engineering. Adv. Drug Deliv. Rev..

[26] Schmitt C., Sanchez C., Desobry-Banon S., Hardy J. (1998). Structure and technofunctional properties of protein-polysaccharide complexes: A review. Crit. Rev. Food Sci. Nutr..

[27] Murray M.J., Snowden M.J. (1995). The preparation, characterisation and applications of colloidal microgels. Adv. Colloid Interface Sci..

[28] Volod’ko A.V., Davydova V.N., Chusovitin E., Sorokina I.V., Dolgikh M.P., Tolstikova T.G., Balagan S.A., Galkin N.G., Yermak I.M. (2014). Soluble chitosan–carrageenan polyelectrolyte complexes and their gastroprotective activity. Carbohydr. Polym..

[29] Volod’ko A.V., Davydova V.N., Nedashkovskaya O.I., Terentieva N.A., Chusovitin E.A., Galkin N.G., Yermak I.M. (2018). Morphology, electrokinetic characteristics and the effect on biofilm formation of carrageenan:chitosan polyelectrolyte complexes. Int. J. Biol. Macromol..

[30] Yermak I.M., Kim Y.H., Titlynov E.A., Isakov V.V., Solov’eva T.F. (1999). Chemical structure and gel properties of carrageenans from algae belonging to the Gigartinaceae and Tichocarpaceae, collected from the Russian Pacific Coast. J. Appl. Phycol..

[31] Wolfrom M., Han T. (1959). The Sulfonation of Chitosan. J. Am. Chem. Soc..

[32] Domszy J., Roberts G. (1985). Evaluation of infrared spectroscopic techniques for analysing chitosan. Die Makromol. Chem..

[33] Volod’Ko A.V., Davydova V.N., Barabanova A.O., Soloveva T.F., Ermak I.M. (2012). Formation of soluble chitosan-carrageenan polyelectrolyte complexes. Chem. Nat. Compd..

[34] Davydova V.N., Volod’ko A.V., Sokolova E.V., Chusovitin E.A., Balagan S.A., Gorbach V.I., Galkin N.G., Yermak I.M., Solov’Eva T.F. (2015). The supramolecular structure of LPS-chitosan complexes of varied composition in relation to their biological activity. Carbohydr. Polym..

[35] Ratanathanawongs Williams S.K., Lee D. (2006). Field-flow fractionation of proteins polysaccharides, synthetic polymers, and supramolecular assemblies. J. Sep. Sci..

[36] Kalim M.D., Bhattacharyya D., Banerjee A., Chattopadhyay S. (2010). Oxidative DNA damage preventive activity and antioxidant potential of plants used in Unani system of medicine. BMC Complement. Altern. Med..

[37] López-Alarcón C., Denicola A. (2013). Evaluating the antioxidant capacity of natural products: A review on chemical and cellular-based assays. Anal. Chim. Acta.

[38] Chen S.K., Tsai M.L., Huang J.R., Chen R.H. (2009). In Vitro Antioxidant Activities of Low-Molecular-Weight Polysaccharides with Various Functional Groups. J. Agric. Food Chem..

[39] Kim K.W., Thomas R.L. (2007). Antioxidative activity of chitosans with varying molecular weights. Food Chem..

[40] Duque G.A., Descoteaux A. (2014). Macrophage cytokines: Involvement in immunity and infectious diseases. Front. Immunol..

[41] Berger J., Reist M., Mayer J.M., Felt O., Gurny R. (2004). Structure and interactions in chitosan hydrogels formed by complexation or aggregation for biomedical applications. Eur. J. Pharm. Biopharm..

[42] Rabson A., Roitt I.M., Delves P.J. (2005). Really Essential Medical Immunology.

[43] Moncada S., Palmer R.M.J., Higgs E.A. (1991). Nitric oxide: Physiology, pathophysiology, and pharmacology. Pharmacol. Rev..

[44] Stuehr D.J., Nathan C.F. (1989). Nitric oxide: A macrophage product responsible for cytostasis and respiratory inhibition in tumor target cells. J. Exp. Med..

[45] Ajisaka K., Agawa S., Nagumo S., Kurato K., Yokoyama T., Arai K., Miyazaki T. (2009). Evaluation and comparison of the antioxidative potency of various carbohydrates using different methods. J. Agric. Food Chem..

[46] Wang J., Hu S., Nie S., Yu Q., Xie M. (2016). Reviews on Mechanisms of In Vitro Antioxidant Activity of Polysaccharides. Oxid. Med. Cell. Longev..

[47] Gouwy M., Struyf S., Proost P., Van Damme J. (2005). Synergy in cytokine and chemokine networks amplifies the inflammatory response. Cytokine Growth Factor Rev..

[48] Du B., Lin C., Bian Z., Xu B. (2015). An insight into anti-inflammatory effects of fungal beta-glucans. Trends Food Sci. Technol..

[49] Wang Y., Zhang L., Li Y., Hou X., Zeng F. (2004). Correlation of structure to antitumor activities of five derivatives of a β-glucan from Poria cocos sclerotium. Carbohydr. Res..

[50] Amann R., Schuligoi R., Lanz I., Donnerer J. (1995). Histamine-induced edema in the rat paw-effect of capsaicin denervation and a CGRP receptor antagonist. Eur. J. Pharmacol..

[51] Sokolova E.V., Kravchenko A.O., Sergeeva N.V., Davydova V.N., Bogdanovich L.N., Yermak I.M. (2020). Effect of carrageenans on some lipid metabolism components in vitro. Carbohydr. Polym..

[52] Dumonde D.C., Maini R.N. (1971). The clinical significance of mediators of cellular immunity. Clin. Exp. Allergy.

[53] Andreis M., Stastny P., Ziff M. (1975). Experimental arthritis produced by injection of mediators of delayed hypersensitivity. Rheum.Annu. Rev..

[54] Bento C.A.M., Cavada B.S., Oliveira J.T.A., Moreira R.A., Barja-Fidalgo C. (1993). Rat paw edema and leukocyte immigration induced by plant lectins. Agents Actions.

[55] Bernfield M., Park P.W., Reizes O., Fitzgerald M.L., Lincecum J., Zako M., Gotte M., Park P.W., Reizes O., Fitzgerald M.L. (1999). Functions of cell surface heparan sulfate proteoglycans. Annu. Rev. Biochem..

[56] Hartig S.M., Greene R.R., Dikov M.M., Prokop A., Davidson J.M. (2007). Multifunctional nanoparticulate polyelectrolyte complexes. Pharm. Res..

[57] Jung T., Kamm W., Breitenbach A., Kaiserling E., Xiao J.X., Kissel T. (2000). Biodegradable nanoparticles for oral delivery of peptides: Is there a role for polymers to affect mucosal uptake?. Eur. J. Pharm. Biopharm..

[58] Klein J. (2007). Probing the interactions of proteins and nanoparticles. Proc. Natl. Acad. Sci. USA.

[59] Tenzer S., Docter D., Kuharev J., Musyanovych A., Fetz V., Hecht R., Schlenk F., Fischer D., Kiouptsi K., Reinhardt C. (2013). Rapid formation of plasma protein corona critically affects nanoparticle pathophysiology. Nat. Nanotechnol..

[60] Liu F., Zhang X., Li Y., Chen Q., Liu F., Zhu X., Mei L., Song X., Liu X., Song Z. (2017). Anti-inflammatory effects of a Mytilus coruscus α-D-glucan (MP-A) in activated macrophage cells via TLR4/NF-κB/MAPK pathway inhibition. Mar. Drugs.

[61] Naberezhnykh G.A., Gorbach V.I., Likhatskaya G.N., Davidova V.N., Solov’eva T.F. (2008). Interaction of chitosans and their N-acylated derivatives with lipopolysaccharide of gram-negative bacteria. Biochemistry.

[62] Barabanova A.O., Shashkov A.S., Glazunov V.P., Isakov V.V., Nebylovskaya T.B., Helbert W., Solov’eva T.F., Yermak I.M. (2008). Structure and properties of carrageenan-like polysaccharide from the red alga Tichocarpus crinitus (Gmel.) Rupr. (Rhodophyta, Tichocarpaceae). J. Appl. Phycol..

[63] Sokolova E.V., Barabanova A.O., Bogdanovich R.N., Khomenko V.A., Solov’eva T.F., Yermak I.M. (2011). In vitro antioxidant properties of red algal polysaccharides. Biomed. Prev. Nutr..

[64] De Groote D., Zangerle P.F., Gevaert Y., Fassotte M.F., Beguin Y., Noizat-Pirenne F., Pirenne J., Gathy R., Lopez M., Dehart I. (1992). Direct stimulation of cytokines (IL-1β, TNF-α, IL-6, IL-2, IFN-γ and GM-CSF) in whole blood. I. Comparison with isolated PBMC stimulation. Cytokine.

[65] Khlebnicova T.S., Piven Y.A., Baranovsky A.V., Lakhvich F.A., Sorokina I.V., Tolstikova T.G. (2019). Fluorine-containing lupane triterpenoid acid derivatives: Design, synthesis and biological evaluation as potential anti-inflammatory agents. Steroids.

[66] Bednarczyk-Cwynar B., Zaprutko L., Marciniak J., Lewandowski G., Szulc M., Kaminska E., Wachowiak N., Mikolajczak P.L. (2012). The analgesic and anti-inflammatory effect of new oleanolic acid acyloxyimino derivative. Eur. J. Pharm. Sci..

